# GEICO1601-ROLANDO: a multicentric single arm Phase II clinical trial to evaluate the combination of olaparib and pegylated liposomal doxorubicin for platinum-resistant ovarian cancer

**DOI:** 10.4155/fsoa-2018-0107

**Published:** 2019-01-10

**Authors:** J Alejandro Perez-Fidalgo, Maria Iglesias, Uriel Bohn, Elisa Calvo, Yolanda Garcia, Eva Guerra, Luis Manso, Ana Santaballa, Antonio Gonzalez-Martin

**Affiliations:** 1 Department of Medical Oncology, Hospital Clinico Universitario de Valencia, CIBERONC, Spain; 2 Department of Medical Oncology, Hospital Universitario Son Llatzer, Palma de Mallorca, Spain; 3 Department of Medical Oncology, Hospital Universitario Dr Negrin, Las Palmas de Gran Canaria, Spain; 4 Department of Medical Oncology, Hospital Universitario Virgen del Rocio, Sevilla, Spain; 5 Department of Medical Oncology, Hospital Universitario Parc Tauli, Sabadell, Spain; 6 Department of Medical Oncology, Hospital Universitario 12 de Octubre, Madrid, Spain; 7 Department of Medical Oncology, Hospital Universitario Ramon y Cajal, Madrid, Spain; 8 Department of Medical Oncology, Hospital Politecnico y Universitario La Fe, Valencia, Spain; 9 Department of Medical Oncology, Clinica Universitaria de Navarra, Madrid, Spain

**Keywords:** maintenance, olaparib, ovarian cancer, pegylated liposomal doxorubicin, platinum-resistant

## Abstract

Response to polyadenosine diphosphate ribose polymerase (*PARP*) inhibitors in platinum-resistant ovarian cancer and in the absence of *BRCA* mutations is very low. Combining PARP inhibitors with other agents might overcome this lack of activity. Here we describe the rationale and design of GEICO1601-ROLANDO (resistant ovarian cancer treated with olaparib and pegylated liposomal doxorubin; NCT03161132). ROLANDO is a Phase II single-arm multicenter trial in which patients are treated with a combination of olaparib and pegylated liposomal doxorubicin (PLD) in platinum-resistant epithelial ovarian, primary peritoneal, or Fallopian tube cancer regardless of the *BRCA* mutation status. The primary end point is progression-free survival at 6 months. Other secondary end points are response rate, disease control rate, quality of life and overall survival.

## Lay abstract

PARP inhibitors as a single agent have shown very modest activity in platinum-resistant ovarian cancer in a BRCA nonselected population. The GEICO1601-ROLANDO trial is a protocol designed with the aim of assessing efficacy and safety of the combination of olaparib and pegylated liposomal doxorubin followed by olaparib maintenance in this setting.

Ovarian cancer is a leading cause of death, being the fourth most frequent cause of mortality in women in developed countries [1].

Family history and the presence of mutations in genes such as *BRCA1* and *BRCA2* significantly increase the risk of development of ovarian cancer; other factors such as hormonal or reproductive factors can either increase or decrease the likelihood of this disease [2].

The overall median survival for ovarian cancer is about 40–50% at 10 years from diagnosis. However, this falls significantly to 21% and <6% when the disease is diagnosed at Stages III and IV, respectively. Approximately 90% of ovarian cancers are epithelial in origin, of which 60–70% are high-grade serous ovarian cancer [3].

Epithelial ovarian cancer is highly chemosensitive and the current standard of care for newly diagnosed cases involves debulking surgery and adjuvant platinum- and taxane-based combination chemotherapy. Although most patients achieve a remission with front-line therapy, unfortunately, up to 80% of patients will relapse [4]. Disease recurring >6 months from the last platinum-based chemotherapy is considered as platinum-sensitive relapse while recurrences within the first 6 months are classified as platinum resistant. Platinum sensitive is more likely to respond to platinum-based chemotherapy; however, the development of platinum resistance is an inevitable event [5].

The development of *PARP* inhibitors for the treatment of tumors deficient in *BRCA1* or *BRCA2* is driven by the concept of synthetic lethality, a phenomenon in which the individual deletion of two independent genes does not cause cell death, but the combined deletion is cytotoxic.

Olaparib (AZD2281) is an oral *PARP*-1 and -2 inhibitor largely tested among ovarian cancer patients. In the Phase I clinical trial patients with different solid tumors refractory to standard treatment were included. Partial response and clinical benefit were observed in those patients harboring BRCA mutation diagnosed with breast, ovarian and prostate cancer [6].

The expansion cohort of this Phase I trial finally included 50 *BRCA* mutation carriers with platinum-refractory (26%), platinum-resistant (48%) and platinum-sensitive relapse (26%) [7]. Olaparib, at different dose levels, achieved a 28% of response rates by response evaluation criteria in solid tumors (RECIST; 95% CI: 16.2–42.5%), 46.2% in platinum-sensitive and 33.5% in platinum-resistant recurrences, with no responses in the platinum-refractory group. Clinical benefit was also meaningful with 46% in the whole series, which was particularly relevant in the platinum-sensitive group (69.2%), but also interesting in the platinum-resistant (45.8%) and -refractory (23.1%) groups.

A later Phase II trial finally confirmed the activity of olaparib in monotherapy in a heavily pretreated *gBRCAmut* ovarian cancer population. In this international study, patients irrespective of interval of platinum were treated after a median of three previous lines in two different cohorts with olaparib 400 mg two-times a day (b.i.d.) and 100 mg b.i.d. Overall response rate (ORR) was 30% in the cohort treated with 400 mg and 13% in the cohort with olaparib 100 mg. The most frequent adverse events were nausea, fatigue and anemia, which were slightly superior in the cohort of 400 mg b.i.d. [8].

In 2012, a multicentre Phase II study by Kaufman *et al*. enrolled individuals with a germline *BRCA1*/2 mutation and recurrent cancer. Eligibility included ovarian cancer resistant to prior platinum; breast cancer with more than or equal to three chemotherapy regimens for metastatic disease; pancreatic cancer with prior gemcitabine treatment; or prostate cancer with progression on hormonal and one systemic therapy. Olaparib was administered as a single agent at 400 mg b.i.d. The primary efficacy end point was tumor response rate. A total of 298 patients received treatment. The tumor response rate was 31.1% in the ovarian cancer cohort (all platinum resistant; Tables 1 & 2) [9].

**Table 1.** **Trials with olaparib in platinum-resistant ovarian cancer harboring BRCA mutations.**

**Study (year) [Ref.]**	**Design**	**n**	**Setting**	**Response rate/clinical benefit rate**	**Platinum-resistant relapse**
Fong *et al*. (2010) [7]	Phase I trial. Expansion cohort of olaparib 400 mg b.i.d. End point: response	50	*BRCA* mutated (41 *BRCA1*, 8 *BRCA2* mutation confirmed, one strong family history)	ORR (CR + PR) 28% (16.2–42.5); CBR 46% (31.8–60.7%)	n = 24 platinum resistant, ORR 33.5% (15.6–55.3), CBR 45.8% (25.6–67.2); n = 13 platinum refractory, ORR 0% (0–24.7), CBR 23.1% (5–53.8)

Kaufman *et al*. (2015) [9]	Phase II (including ovarian, breast, pancreas, prostate and other cancers). End point: response	218 Ovarian cancer 193	*BRCA*-mutated ovarian cancer: 148 *BRCA1* 44 *BRCA2* 1 *BRCA1* and *BRCA2*	ORR 26.2% (21.3–31.6)	n = 193, ORR 31.1% (24.6–38.1)

b.i.d.: Two-times a day; CBR: Clinical benefit rate; CR: Complete response; ORR: Overall response rate; PR: Partial response.

**Table 2.** **Trials with olaparib in platinum-resistant ovarian cancer irrespectively of BRCA mutation status.**

**Study (year) [Ref.]**	**Design**	**n**	**Setting**	**Response rate in overall population**	**Response rate in *BRCA*-mutated population**	**Response rate in *BRCA* wt population**
Gelmon *et al*. (2011) [10]	Phase II single arm (olaparib 400 b.i.d.). End point: response	91 (65 ovarian cancer)→17 *BRCA* mut and 47 *BRCA* no mut	Recurrent ovarian cancer. Median 3 previous lines (1–10)	OR 29%	60% (3/5 platinum sensitive); 33% (4/12 platinum resistant)	50% (10/20 platinum sensitive); 4% (1/26 platinum resistant)

Based on the study by Kaufman *et al*., olaparib was approved by the US FDA for refractory, advanced ovarian cancer associated with germline breast cancer susceptibility gene (*BRCA*) mutations in 2014. In August 2017, olaparib was additionally approved by the FDA as a maintenance therapy for women with recurrent epithelial ovarian, fallopian tube or primary peritoneal cancer who have achieved complete or partial response to platinum-based chemotherapy, irrespective of their *BRCA* status.

However, EMA approval of olaparib has been limited to platinum-sensitive relapse.

Olaparib was compared with pegylated liposomal doxorubicin (PLD) in a multicenter randomized Phase II trial. Patients with BRCA1 or BRCA2 germline mutations recurring within 12 months of prior platinum therapy were randomized to receive olaparib 200 mg b.i.d. versus olaparib 400 mg b.i.d. versus PLD 50 mg/m^2^ intravenously every 28 days [11]. 97 patients were included, of whom 42.4–56.4% were platinum resistant. Of note, the proportion of platinum-resistant relapses was inferior in the PLD arm (42.4%). Progression-free survival (PFS) was 6.5 months (95% CI: 5.5–10.1 months), 8.8 months (95% CI: 5.4–9.2 months) and 7.1 months (95% CI: 3.7–10.7 months) for olaparib 200 mg, olaparib 400 mg and PLD, respectively, with an unexpectedly high PFS for the PLD arm. There were no statistically significant differences between combined arms of single agent olaparib versus PLD (HR: 0.88; 95% CI: 0.51–1.56; p = 0.66). RECIST-assessed response rates were 25, 31 and 18%, respectively, with no statistically significant differences. This study showed a similar activity and a trend toward improvement with olaparib versus PLD in patients with relapsed ovarian cancer and *BRCA1* or *BRCA2* mutations.

Although olaparib has been tested in combination to chemotherapy with carboplatin and paclitaxel in a randomized Phase II trial [12], the only previous experience of a combination of PLD and concurrent olaparib comes from a Phase I trial [13].

## The ROLANDO trial

Here we describe the rationale and design of ROLANDO, a single-arm Phase II trial, designed to evaluate the efficacy and safety of the combination of olaparib plus PLD in platinum-resistant epithelial ovarian cancer.

### Background & rationale

The combination of olaparib and liposomal doxorubicin in ovarian cancer has been tested previously in a Phase I trial [13]. 44 patients with ovarian (n = 28), breast (n = 13) and other solid tumors (n = 3) were treated with olaparib continuously (days 1–28) or intermittently (days 1–7) plus PLD (40 mg/m^2^ day 1). Seven olaparib cohorts were explored (50–400 mg b.i.d.) to determine the recommended dose. Olaparib 400 mg b.i.d. plus PLD was well tolerated and no dose lethal toxicity was detected. Grade 3 adverse events (AE) were reported in 27 patients (61%), and serious AE occurred in 12 patients (27%). Most frequent Grade 3 AE were stomatitis, nausea and asthenia, which happened mainly in the cohort of olaparib 400 mg b.i.d. continuously.

No major interactions were observed between PLD and olaparib. The ORR was 33% (14 out of 42 patients, three complete responses). A total of 13 responders had ovarian cancer (response rate 46.4% in ovarian cancer), of whom ten were platinum sensitive.

Recently, a preclinical study demonstrated a synergism of the combination of olaparib with doxorubicin in 2D and spheroid models of ovarian cancer [14]. This study showed that DNA damage was increased at a molar concentration of doxorubicin: olaparib of 50:1 in both *BRCA* mutant and *BR CA* wild type cell lines UWB1-289. The combination of PLD and olaparib resulted in a significantly increased number of double-strand breaks assessed by γ *H2AX* assay compared with olaparib single agent (approximately 10- and 13-fold increases for UWB1.289 and UWB1.289 +*BRCA*, respectively) and to doxorubicin single agent (approximately 50 and 65% increases, respectively).

### Design

#### Study design

GEICO 1601-ROLANDO (NCT03161132) is a single-arm prospective trial to assess the efficacy and safety of the combination of olaparib and PLD in the platinum-resistant setting.

#### Primary & secondary objectives

Primary efficacy end point is 6 months PFS rate (PFS6m) of the combination of olaparib 300 mg b.i.d. in tablet formulation + PLD 40 mg/m^2^ intravenously every 28 days followed by olaparib 300 mg b.i.d. tablet formulation in platinum-resistant relapsed ovarian cancer patients.

Secondary end points are safety and objective response rate (ORR), defined as the sum of complete and partial responses by RECIST 1.1, disease-control rate (complete response, partial response or stable disease), CA-125 response, PFS, postprogression survival, overall survival, quality of life and growth-modulation index.

#### Key eligibility criteria

Key eligibility criteria are shown in Table 3.

**Table 3.** **Key inclusion and exclusion criteria.**

**Key inclusion criteria**	**Key exclusion criteria**
Platinum-resistant recurrent high-grade serous or endometrioid ovarian cancer, primary peritoneal or fallopian tube cancer (>28 days and <6 months since previous platinum)	Platinum-refractory ovarian cancer (relapse during or <28 days since last platinum-containing chemotherapy)

≤3 prior chemotherapy regimens	Previous therapy with *PARP* inhibitor

Any *BRCA* status: – *BRCA* wt or unknown: at least one previous platinum-sensitive relapse – *BRCA* mut: primary platinum resistance is allowed	Previous therapy with PLD except if > 6 months since last PLD and given as a part of a platinum-sensitive relapse

ECOG ≤2	LVEF less than threshold of normality

Measurable disease according to RECIST 1.1	

Adequate renal, liver and bone marrow function (Hb ≥10 g/dl)	

ECOG: Eastern Cooperative Oncology Group; Hb: Hemoglobin; LVEF: Left ventricular ejection fraction; PLD: Pegylated liposomal doxorubicin; RECIST: Response evaluation criteria in solid tumors.

#### Planned sample size

The sample size was determined based on exact single-stage Phase II design. We assumed that a new combination with 6-month PFS of 15% or less would be considered of little clinical significance and that an agent with PFS 40% or higher would be of interest for further investigation. With 90% power and at a two-sided 0.05 significance level, 27 evaluable patients should be included in the study, requiring at least ten patients surviving after 6 months to reject the null hypothesis.

In order to obtain the required evaluable number of patients we expect to recruit 32 patients (20% dropouts).

#### Dose & schedule of therapy

**Figure 1.**
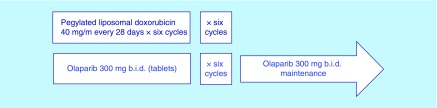
**Treatment schedule of the GEICO1601-ROLANDO trial.** b.i.d.: Twice daily.

This is a single arm trial in which patients will be treated with a combination of PLD and olaparib for six cycles following maintenance therapy with olaparib (Figure 1).

#### Efficacy evaluation

For efficacy assesment subsequent CT scan tumor evaluation according to RECIST 1.1 criteria will be performed systematically every 8 weeks. Tumor assessment will also be done at planned intervals. Visits will include systematically only physical examination and CA-125 analysis.

Patients must have been treated with at least two cycles in order to be considered evaluable. After six cycles of PLD plus olaparib, the patient will be assessed for response to treatment, and only those patients achieving response or stable disease will go on to the maintenance phase.

Disease follow-up will be done according to RECIST criteria V1.1, computed tomography scans, which should be performed at screening and every 8 weeks during the first 12 months and every 12 weeks after 12 months from start of treatment, both until disease progression.

Also, CA-125 levels will be followed, CA-125 determinations should be performed at screening and every 8 weeks during the first 12 months and every 12 weeks after 12 months from start of treatment, both until disease progression. Rising of CA-125 will not be considered progression until availability of confirmatory computed tomography scan.

#### Safety evaluation

Patients will be visited according the following schema:

- Every week during the first month of treatment.
- Every 2 weeks during the second month of treatment.
- Every 4 weeks from 3 up to 12 months (calculated from first study treatment administration).
- Every 12 weeks up to 2 years calculated from first study treatment administration.
- According to local normal clinical practice from 2 years until exit.
- At the end of study treatment.
- Safety visit (after 30 days of end of treatment).

AE will be graded according to National Cancer Institute – Common Terminology Criteria for Adverse Events (NCI CTCAE) V4.03. AE G≥2 will be recorded at each visit during treatment until the safety visit.

Safety visit will be performed at day 30 +/-3 days after end of treatment in order to determine patient general status.

#### Statistical analysis

Demographics and baseline characteristics will be summarized using descriptive statistics. For categorical variables, the number (n) and percent of each category within a parameter will be presented. For continuous variables, the sample size (n), mean, median and standard deviation, as well as the minimum and maximum values, will be determined.

All efficacy analyses will be based primarily on the intention-to-treat analysis set and secondarily on the per protocol analysis set, which are defined as follows:

- Intention-to-treat analysis set: all patients participating in the study receiving at least a single dose of study medication.
- Per protocol analysis set: all patients participating in the study who fulfill the protocol in terms of the eligibility, interventions and outcome assessment receiving at least two cycles of DLP + olaparib.

The primary end point of PFS6m will be estimated using Kaplan–Meier method.

The secondary end points of ORR defined as the proportion of patients with a confirmed complete response or partial response as per RECIST 1.1, and the disease control rate defined as the sum of complete response + partial response + stable disease will be estimated along with a 95% CI.

Additional secondary end points of PFS, postprogression survival and overall survival (as assessed by the investigator) will be estimated using Kaplan–Meier method.

#### Recruiting sites

The study is enrolling patients in eight institutions in Spain.

## Conclusion

The GEICO 1601-ROLANDO trial is a study that aims to respond to the question of whether the combination of PLD and olaparib has a role in the platinum-resistant setting irrespective of *BRCA* mutation status.

**Executive summary**

- *PARP* inhibitors (PARPi) have shown important activity in platinum-sensitive relapse and BRCA-mutated ovarian cancer. However, activity of PARPi is modest in platinum-resistant relapse and *BRCA*-unknown or not mutated patients.
- The GEICO1601-ROLANDO trial is a single-arm Phase II trial designed to evaluate the efficacy and safety of the combination of olaparib and pegylated liposomal doxorubicin (PLD) in platinum-resistant ovarian cancer irrespectively of BRCA mutation status.
- Primary end point is progression-free survival (PFS) at 6 months.
- Secondary end points are safety, overall response rate, disease-control rate, CA-125 response, PFS and overall survival.
- Among the key inclusion criteria are high-grade serous or endometrioid ovarian carcinoma, platinum resistance relapse defined as >28 days and <6 months since last platinum-containing schedule, no more than three previous lines, if *BRCA* status is unknown or nonmutated at least one previous platinum-sensitive relapse is required, if *BRCA* is mutated primary platinum resistance is allowed, an Eastern Cooperative Oncology Group less than or equal to two, measurable disease and appropriate bone marrow and renal and hepatic function. Previous PLD is allowed if given during a platinum-sensitive relapse and > 6 months. Previous PARPi is excluded.
- PLD will be administered at 40 mg/m^2^ every 28 days in combination with olaparib tablets 300 mg/12 h continuously for six cycles followed by maintenance with olaparib. The planned sample size was calculated assuming that PFS6m 15% or less will be clinically futile and that PFS6m 40% or higher is clinically relevant. Sample size needed with 20% dropouts is 32 patients.
- To date 16 patients have been recruited; results are expected for the end of 2019.
